# A Review of Persuasive Principles in Mobile Apps for Chronic Arthritis Patients: Opportunities for Improvement

**DOI:** 10.2196/mhealth.6286

**Published:** 2016-10-13

**Authors:** Jonas Geuens, Thijs Willem Swinnen, Rene Westhovens, Kurt de Vlam, Luc Geurts, Vero Vanden Abeele

**Affiliations:** ^1^ e-Media Lab KU Leuven Leuven Belgium; ^2^ UZ Gasthuisberg Division of Rheumatology University Hospitals Leuven Leuven Belgium; ^3^ Skeletal Biology and Engineering Research Center Department of Development and Regeneration KU Leuven Leuven Belgium

**Keywords:** persuasive technology, mobile applications, chronic arthritis

## Abstract

**Background:**

Chronic arthritis (CA), an umbrella term for inflammatory rheumatic and other musculoskeletal diseases, is highly prevalent. Effective disease-modifying antirheumatic drugs for CA are available, with the exception of osteoarthritis, but require a long-term commitment of patients to comply with the medication regimen and management program as well as a tight follow-up by the treating physician and health professionals. Additionally, patients are advised to participate in physical exercise programs. Adherence to exercises and physical activity programs is often very low. Patients would benefit from support to increase medication compliance as well as compliance to the physical exercise programs. To address these shortcomings, health apps for CA patients have been created. These mobile apps assist patients in self-management of overall health measures, health prevention, and disease management. By including persuasive principles designed to reinforce, change, or shape attitudes or behaviors, health apps can transform into support tools that motivate and stimulate users to achieve or keep up with target behavior, also called persuasive systems. However, the extent to which health apps for CA patients consciously and successfully employ such persuasive principles remains unknown.

**Objective:**

The objective of this study was to evaluate the number and type of persuasive principles present in current health apps for CA patients.

**Methods:**

A review of apps for arthritis patients was conducted across the three major app stores (Google Play, Apple App Store, and Windows Phone Store). Collected apps were coded according to 37 persuasive principles, based on an altered version of the Persuasive System Design taxonomy of Oinas-Kukkonen and Harjuma and the taxonomy of Behavior Change Techniques of Michie and Abraham. In addition, user ratings, number of installs, and price of the apps were also coded.

**Results:**

We coded 28 apps. On average, 5.8 out of 37 persuasive principles were used in each app. The most used category of persuasive principles was System Credibility with an average of 2.6 principles. Task Support was the second most used, with an average of 2.3 persuasive principles. Next was Dialogue Support with an average of 0.5 principles. Social Support was last with an average of 0.01 persuasive principles only.

**Conclusions:**

Current health apps for CA patients would benefit from adding Social Support techniques (eg, social media, user fora) and extending Dialogue Support techniques (eg, rewards, praise). The addition of automated tracking of health-related parameters (eg, physical activity, step count) could further reduce the effort for CA patients to manage their disease and thus increase Task Support. Finally, apps for health could benefit from a more evidence-based approach, both in developing the app as well as ensuring that content can be verified as scientifically proven, which will result in enhanced System Credibility.

## Introduction

Chronic arthritis (CA) is an umbrella term for inflammatory rheumatic and musculoskeletal diseases such as rheumatoid arthritis (RA), osteoarthritis (OA), and spondyloarthritis (SpA). CA is highly prevalent. One in five adults in the United States has doctor-diagnosed arthritis [1]. CA is typically associated with processes of inflammation and/or destruction, which cause joint pain, swelling, stiffness and instability, joint destruction, or bony ankylosis resulting in progressive immobility [2]. These disease processes largely contribute to limitations in performing day-to-day activities such as walking, cleaning, and working [3]. Luckily, effective disease-modifying antirheumatic drugs have become available to tackle RA and SpA. However, they require the patient’s long-term commitment to tightly follow up on disease parameters by the treating physician and other health professionals, as well as compliance with the medication regimen and other therapy proposals [4,5]. Treatment recommendations for all CA also include physical therapy programs in order to improve aspects of physical fitness such as cardiovascular endurance, muscle strength, posture and movement control, range of motion, and balance [3,6-8]. Ample evidence suggests that physical exercise has strong benefits [9-11]. Unfortunately, between 35% and 75% of patients with CA fail to adhere to the physical exercise recommendations of their therapist [12-14]. Besides arthritis-specific barriers (eg, pain, disability), adherence to therapy is reduced by personal barriers (eg, lack of motivation) and contextual barriers (eg, lack of skilled staff and facilities) [15,16]. Clearly there is a need to support patients in managing their disease and adhering to their physical exercise programs.

Perhaps mobile health (mHealth) apps can be part of a solution for supporting CA patients. Health apps are defined as “mobile applications that assist consumers in self-management of overall wellness, disease prevention and disease management” [17]. These apps are further advancements of the telehealth movement, not only supporting remote interventions but also providing the opportunity to intervene at any time and place [18]. mHealth apps provide clear benefits for treatment, assessment, and self-management of CA [19], for example, the assessment of gait in rheumatic diseases [20] or the logging of pain and physical condition [21]. The advent of health apps is strongly interlinked with the rise of mobile phones, specifically smartphones. Smartphone penetration rate at the end of 2015 was predicted to be 42.6% on a global scale, 59.8% in the United States, and 54.9% in Western Europe [22-24]. Most patients today already own and use smartphones on a daily basis. Many CA researchers have identified clear needs and opportunities for mobile phone apps to tackle educational, lifestyle, and treatment interventions to ease delivery and increase involvement of CA patients [19-21,25].

In addition, there has been a call for a more conscious design of these apps, starting from the CA patients’ needs and addressing CA patients’ motivations for using health apps [26-28]. In particular, designing for patients who suffer from comorbidities involving chronic pain, depression, and fatigue presents specific challenges [29-31] with respect to sustained motivation. In this study, we want to investigate how to increase the motivation of CA patients to use mHealth apps, and more particularly by including “persuasive principles.” Persuasive principles are specific design techniques such as offering praise, providing reminders, imitating social agents, providing “social support,” or augmenting “system credibility” [32]. By including persuasive principles, health apps can transform into supportive tools that motivate and stimulate users to achieve or keep up with targeted behavior, also called persuasive systems, defined as “computerized software or information systems designed to reinforce, change or shape attitudes or behaviors or both without using coercion or deception” [33].

It has been argued that the design of current health apps lacks a “conscious” implementation of persuasive principles [27,29]. According to Kelders et al [33] and Tomlinson et al [34], apps in health care are often designed and treated as “black boxes”: the technology works but the design itself is not evidence-based nor based on behavior change. This may result in technology that has a low impact on health care practices [35-37].

In this paper, we investigated which persuasive principles are most prevalent in current apps directed at CA management and which principles are lacking. This knowledge may inform and inspire health professionals who are looking for innovative ways to support their interventions with mHealth apps. It is our aspiration that this analysis can help health experts select better quality apps to help patients manage their disease in a better and more effective way.

This insight may equally help health professions and app developers build more effective support tools. Particularly, this knowledge may help how to move beyond the current state and result in mHealth apps that are more motivating for CA patients and hence more effective with respect to, for example, adherence of therapies or the removal of contextual barriers.

Several theories, frameworks, and taxonomies exist to guide designers of persuasive systems. Oinas-Kukkonen and Harjumaa proposed a model of Persuasive Systems Design (PSD) [32]. This model contains 28 persuasive system design techniques divided over four categories: primary task support, dialogue support, social support, and system credibility support. Primary task support aims to persuade the user to complete a task by supporting the user in their execution of the task. This category includes, for example, reducing one complex task into a set of smaller tasks that are easier to complete or tunneling to offer the user a trajectory. Dialogue support provides system feedback to guide the user towards the intended behavior. Examples include providing rewards or praise to the user and providing reminders. Social support strengthens the overall persuasiveness of a software system by leveraging the human nature to interact with others [38,39]. Examples include comparing oneself to the norm of a population, cooperating with other users to achieve a similar goal, and learning behaviors or actions by observing others. Finally, system credibility support includes principles on how to design a system so that it is more credible and thus more persuasive.

Besides the PSD model by Oinas-Kukkonen and Harjumaa, another established framework is the one offered by Abraham and Michie. These authors proposed a Taxonomy of Behavior Change Techniques (BCT) [40]. Different from the PSD model, the 26 BCT principles are not organized in broader categories, but rather the authors link the principles explicitly to the underlying theoretical models of behavior change such as Operant Conditioning [41], Social Cognitive Theory [42], and Theory of Planned Behavior [43].

Several authors used either the PSD model or BCT to study Web-based health interventions or a combination of both [44-48] to study the effect on motivation or adherence to the intervention. Kelders et al [44] conducted a systematic review of Web-based health interventions, relying on the PSD model, to determine key persuasive factors for adherence to the intervention. They classified which persuasive system design principles were most prevalent and how they impacted adherence to the intervention. They found that primary task support showed the highest mean, while social support showed the lowest mean. However, while primary task support principles were most commonly employed in Web-based interventions, they did not show any predictive value for adherence. In contrast, social support principles showed a significant contribution to better adherence, yet social support principles were least implemented in Web-based interventions. Overall, Kelders et al concluded that the use of persuasive technology elements can explain a significant amount of the variance in adherence.

Lehto et al [45] used the PSD principles as well, to classify Web-based alcohol and smoking interventions. Again, they found primary task support components (eg, reduction of task complexity and self-monitoring) to be widely utilized and reported on widely in the reviewed studies. However, they found a lack of tailoring, which may imply that the interventions are not targeting a specific audience. The conclusion was that more research is needed to increase the understanding of persuasive principles in interventions and their contributions to intervention outcomes.

Overall, these studies found that interventions that incorporated more BCTs also tended to have larger effects as compared to interventions that incorporated fewer BCTs, and that different techniques are beneficial for different types of intended behaviors. In particular, social support principles show significant contribution to adherence, yet they are least implemented.

To date, only a few studies have been conducted on persuasive principles in health apps [49-52]. As mentioned, the ubiquity and pervasiveness of mobile phones enable health interventions to go beyond what can be offered via a Web-based intervention. Additional sensing and networking features bring along opportunities for real-time interactions and monitoring, context awareness (eg, localization), physical activity sensing, etc. It is therefore interesting to investigate whether persuasive principles are also used in health apps on smartphones.

Matthews et al conducted a systematic review of persuasive principles in mobile apps promoting physical activity [52]. They also used the PSD model of Oinas-Kukkonen and Harjumaa and reviewed 20 articles describing the use of mobile apps to promote physical activity. The authors found on average only 4 PSD principles (out of 28) were implemented. The most common PSD principle was self-monitoring (implemented in 14 of 20 analyzed systems), a feature that supports a user’s primary task and that was not listed as an important principle in the previous Web-based interventions. In addition, apps frequently implemented a variety of dialogue support and social support principles, as a combination of praise, rewards, reminders, and suggestion to motivate the user to be physically active. They found the most lacking category to be system credibility, in other words, PSD principles that influence the way a user perceives the credibility of the system. Hence, Matthews et al concluded that it is “not clear for users to judge the extent to which the apps are credible or not.”

In line with the previous authors, Vollmer et al launched a call for “Apps seeking theories” [50]. Vollmer et al conducted a systematic review of BCTs in cancer survivorship apps. They identified 68 apps for cancer survivorship on both the Android and iOS platform. Interestingly, these authors developed a coding manual specifically for mHealth apps for cancer survivorship, based on both the BCT taxonomy and PSD model. This coding manual contained 17 persuasive principles. Vollmer et al found that their 68 apps on average contained 4 persuasive principles. They highlighted as well as Matthews et al that a more theory-based approach is needed when designing and developing mHealth apps, especially with respect to the persuasive design/behavior techniques that could empower behavior change: “While the current advancements in mobile hardware, sensors and electronic patient records offer opportunities for health apps, a better understanding is needed of how this translates into benefits for patients” [50].

There is a clear need for health apps to provide treatment, assessment, and self-management of chronic arthritis [19-21,25]. However, currently, these apps lack a conscious implementation of persuasive principles [27], [29]. To contribute to the knowledge surrounding persuasive principles in health apps for chronic arthritis, this paper investigates to what extent persuasive principles are found in disease management apps for arthritis and whether these persuasive principles contribute towards a higher rating of apps. It is our hope that this analysis can help health experts select better quality apps to help patients manage their disease in a better and more effective way. This insight may equally help app developers and health professionals to build more effective support tools. CA patients may in turn benefit from newly developed apps that are more motivating and effective.

## Methods

### Eligibility Criteria for Health Apps

A review of health apps (ie, target intervention) aimed at the management of chronic arthritis (ie, target population) was conducted. This review included all apps available on Google Play (Google, Mountain View, CA), the Apple App Store (Apple Inc, Cupertino, CA), and the Windows Phone Store (Microsoft, Redmond, WA) between January 15 and June 15, 2015. Criteria for inclusion were that the app (1) targets one or more arthritis-related diseases, (2) aids in the management of arthritis-related disease factors, and (3) targets adult patients. Because Google Play employs a broad-spectrum algorithm to return apps related to the search, a number of apps related to arthritis but not intended for patients with arthritis were excluded. Exclusion criteria were (1) apps intended for medical personnel only, (2) magazines, general fitness or pain trackers, or food guides in app format, and (3) apps that failed to install or open on our test devices. These exclusion criteria were applied during the initial screening. There were no language restrictions.

### Search Criteria and Procedures

The used search terms included a range of words related to arthritis as a disease. All three app stores were searched using the same keywords: Arthrit*, Rheuma*, Spondyl*, Osteo*, Bechterew. The search was conducted independently by 2 researchers (JG, VVA). In case one reviewer included an app that was not recorded by the other reviewer, that app was added to the full list to be coded. This was done due to the rapidly changing content of the app stores where new apps are added every day and because both reviewers searched the app stores at different moments.

**Figure 1:**
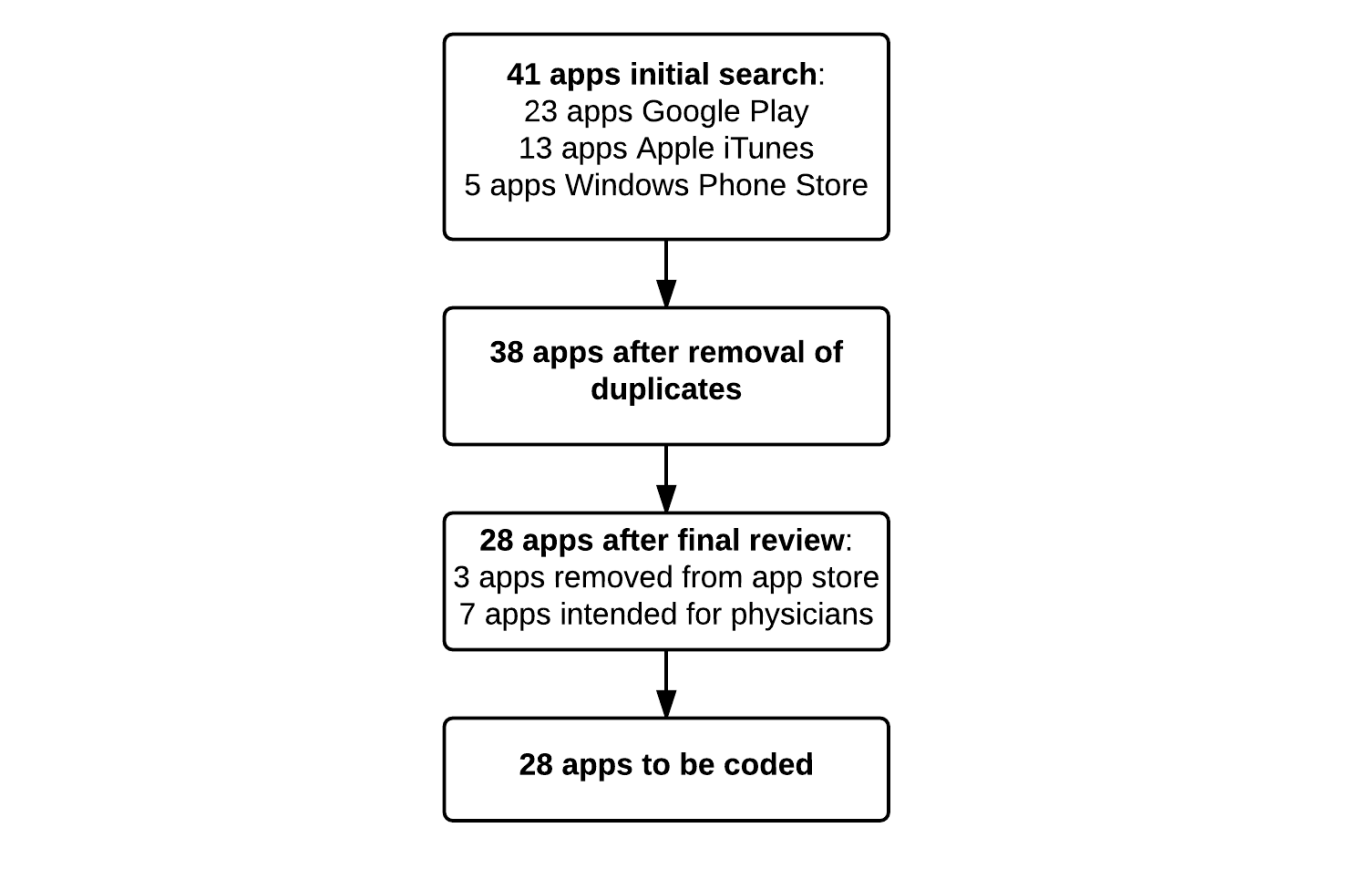
App selection flow.

Because the search algorithm of Google Play continues to load new content related to the search terms, the initial selection stopped when the last 10 app titles were no longer relevant to our search. An initial screening revealed 41 relevant apps across all three app stores. This initial screening was done using keywords described above and by reviewing screenshots and app descriptions. Only apps that met the inclusion criteria were selected, for example, apps that were clearly intended for medical personnel were excluded from the initial selection. After removing duplicates present in one or more of the app stores, 38 apps were left to be screened further. See Figure 1 for the app selection flow.

In the next screening step, each app was installed on a mobile device, either an LG Nexus 4 (LG Electronics, Seoul, South Korea) running Android 5.0.2 (Google) or an iOS (Apple Inc) tablet, running iOS version 8, or a Windows 8 (Microsoft) phone. If multiple platforms were available, the Android platform was chosen first, next iOS, and Windows Phone as a third platform. Each app was then used and tested until a good understanding of the features of the app was established. This was done to determine features not explained in the description of the app. During this screening phase, seven more apps were identified that were intended for therapists or physicians only and not patients. These apps did pass the initial screening (based on their title and screenshots) but were found to meet the exclusion criteria (ie, intended for medical personnel only) after further analysis. Finally, three more apps were removed from the app store by the developers between searching and coding. These were excluded as well. A total of 28 apps remained to be coded.

### Data Extraction and Analyses

Apart from the behavior change/persuasive design principles (see coding system below), the name, targeted disease, platform(s), price, language, average user rating, number of user ratings, number of installs, and finally the last update were coded for every app.

The average user rating and the number of user ratings were also logged since they can provide a good indication of how the app was valued by its users. However, requiring a minimum of user ratings is good practice in order to limit the influence of biased ratings (eg, user ratings that are influenced by the app owners/developers). Therefore, the average user rating of apps was logged only when it had user ratings coming from at least 5 different users. This is also in line with the Apple App store that also sets the minimum number of user ratings necessary for a rating to appear on five. In summary, 14 apps had sufficient user ratings and 14 apps did not show a number of user ratings above the required threshold of five.

In addition, for the apps originating from the Google Play Store, the range of installs was also logged. The Google Play Store does not provide the exact number of installs but rather provides a general indication according to the following brackets: 1-10, 11-50, 50-100, 100-500, 500-1000, 1000-5000, 5000-10000, 10000-50000, etc. Unfortunately, the number of installs is not available to the public in the Apple App Store or the Windows Phone Store.

Finally, to estimate the effects of the number of implemented persuasive principles on favorability of apps, a correlation analysis was performed between the number of persuasive principles and the number of user ratings as well as the number of installs and the average user rating.

### Coding System for Persuasive Design

Apps were coded for persuasive design principles according to an adaptation of the 28 PSD principles by Harjumaa and Oinas-Kukkonen [32] and the Taxonomy of Behavior Change Techniques by Abraham and Michie [40] composed of 26 BCTs. Principles that were ambiguous (eg, “Liking,” being visually attractive) or overlapped with other principles within the same taxonomy (PSD or BCT) (eg, “trustworthiness” and “surface credibility”) were merged, hence ending up with 37 principles organized according to the four overarching categories of task support, dialogue support, system credibility, and social support. Prior to coding, both coders discussed every principle and provided an app-related example to minimize misconceptions.

For each app, the specific and total amount of behavior change/persuasive design principles used was coded and calculated by 2 independent coders (JG and VVA) and a third (JH) in case of disagreement. Agreement occurs when 2 coders both mark the presence or absence of a principle. Disagreement occurs when one coder marks the presence of a principle while the other marks an absence. Intercoder reliability was calculated using both Cohen’s kappa statistic and as percentages of agreement. Reliability values were found to be between 71.4 and 100% wise agreement and between .65 and 1.0 kappa agreement depending on the principle. The mean value of all kappa scores was .96 (SD .08). All kappa values were significant at *P*<.0005.

The lowest rated principle in terms of intercoder reliability was surface credibility (percentage wise agreement: 96.4, kappa agreement: .65) and micro tailoring (percent wise agreement: 96.4, kappa: .781). The first achieved a low kappa value due to a very high percent wise agreement, high level of occurrence, and chance correction used by the kappa calculation. Overall, all intercoder reliability values are at an acceptable level (ie, >.60 [53,54]) with 26 principles (out of 37) scoring perfect agreement. Intercoder reliability scores for each principles can be found in the Results section.

## Results

As mentioned, 28 apps (see Table 1) met the inclusion criteria and were reviewed according to the combination of the behavior change/persuasive design principles.

**Table 1:** Detailed description of the 28 health apps that met the selection criteria.

Name	Targeted disease^a^	Platform^b^	Installs, n	Rating (out of 5)^c^	Ratings, n	Price, US $	Language	Persuasive principles, n
ArthritisID	A	iOS				0.00	English	13
Arthritis Diary	A	An/WP				4.99	English	10
NHS 24 MSK Help	MSK	iOS				0.00	English	10
RA Patient Companion	RA	iOS				0.00	English	10
Pauseboogie fra Gigtforeningen	A	An/iOS	5000-1000	4.0	39	0.00	Danish	8
Rheuma AKTIV	RA	iOS				0.00	German	8
Andar	RA	An	100-500	3.3	3	0.00	Spanish	7
MyRA	RA	iOS		4.5	62	0.00	English	7
RA Helper	RA	An	500-1000	4.8	5	0.00	English/ Slovenian	7
RAPA – RA betegalkalmazás	RA	An	50-100	4.8	5	0.00	Hungarian	7
RheumaTrack RA	RA/SpA	An/iOS	10,000-50,000	4.2	375	0.00	English	7
Track + React	A	iOS		3.4	72	0.00	English	7
Back to Action	SpA	An/iOS	1000-5000	4.2	51	0.00	English	6
Bewegen met Bechterew	SpA	An	100-500	2.1	8	0.00	Dutch	6
iAnkylosing Spondylitis	SpA	iOS				0.00	English	6
DAS Calculadora	RA	An	10-50			0.00	Spanish	5
RADAI	A	WP				0.99	English	5
Arthritis Symptoms+Treatment	RA	An	500-1000	4.5	31	0.00	English	4
RheumaHelper	RA	An/iOS	5000-10,000	4.3	157	0.00	English	4
RhEumAtic Disease activitY	RA	iOS				0.00	English	4
Arthritis Relief	A	WP		5.0	1	4.99	English	3
DAS28 - RA	RA	An	5000-10,000	4.1	64	0.00	English	3
DAS28 Free	RA	An	1000-5000	3.6	8	0.00	English	3
Juvenile RA	RA	An	10-50	4.5	2	0.00	English	3
Living Well With Arthritis	A	iOS				0.99	English	3
Rheumatoid Arthritis Disease	RA	An	100-500	4.4	11	0.00	English	3
Rheumatoid Arthritis of Knee	RA	An	5000-10,000	4.0	47	0.00	English	3
Arthritis	A	WP				3.99	English	1

^a^A: arthritis, MSK: musculoskeletal diseases, RA: rheumatoid arthritis, SpA: spondyloarthritis.

^b^An: Android, WP: Windows phone.

^c^Rating from 1-5 with 1 being the lowest rating.

**Table 2:** Persuasive design principles and number of apps (N=28) that used the principle.

Design principles		Apps, n	Ƙappa (percentage of agreement)
**Task support**			
	General information^a^	16	.786 (89.3)
	Self-monitoring	12	.926 (96.4)
	Reduction	9	.836 (92.9)
	Logging^a^	9	.92 (96.4)
	Instruction^a^	7	.909 (96.4)
	Goal setting	3	1.0 (100)
	Micro tailoring^a^	3	.781 (96.4)
	Macro tailoring^a^	1	1.0 (100)
	Simulation	1	1.0 (100)
	Contextual cues^a^	1	1.0 (100)
	Tunneling	0	1.0 (100)
	Tracking^a^	0	1.0 (100)
	Rehearsal	0	1.0 (100)
	Behavioral contract^a^	0	1.0 (100)
**Dialogue support**			
	Reminders	10	1.0 (100)
	Suggestion	2	1.0 (100)
	Rewards	1	1.0 (100)
	Praise	0	1.0 (100)
	Similarity	0	1.0 (100)
	Personalization	0	1.0 (100)
	Social role	0	1.0 (100)
	Prompt self-talk^a^	0	1.0 (100)
**System credibility**			
	Surface credibility	26	.65 (96.4)
	Expertise	25	.837 (71.4)
	Verifiability	8	1.0 (89.3)
	Real-world feel	7	.884 (85.7)
	Authority	7	.837 (96.4)
	Third-party endorsements	0	1.0 (100)
**Social support**			
	Social interaction^a^	1	1.0 (100)
	Social learning	0	1.0 (100)
	Social identification^a^	0	1.0 (100)
	Social comparison	0	1.0 (100)
	Normative influence	0	1.0 (100)
	Social facilitation	0	1.0 (100)
	Cooperation	0	1.0 (100)
	Competition	0	1.0 (100)
	Recognition	0	1.0 (100)

^a^Principles added from behavior change techniques.

### Prevalence of Behavior Change/Persuasive Design Principles

**Figure 2:**
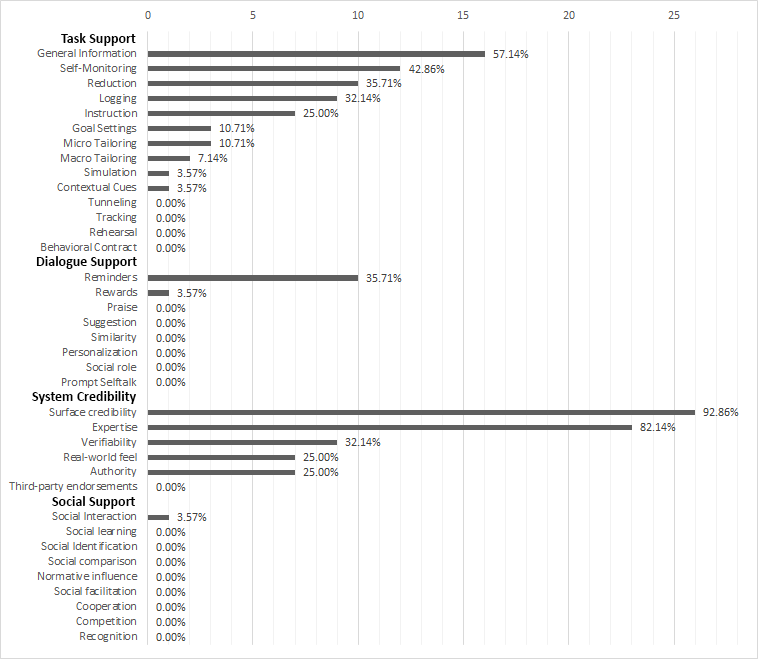
Prevalence of behavior change/persuasive design categories and principles.

On average, an app was found to utilize 5.8 (median 6) behavior change/persuasive design principles out of 37. The maximum number of principles used in one app was 10. The least number of principles used was 1 (out of 37) in one app. Of the four behavior change/persuasive design categories, the most used was system credibility with an average of 2.6 (median 2) principles (ie, on average, each app used 2.6/37 principles from the system credibility category), then task support with an average of 2.3 (median 2) principles, next dialogue support with an average of 0.5 (median 0) principles, and finally social support with an average of 0.01 (median 0) (see Figure 2).

Analyzing the principles within each behavior change/persuasive design category more carefully, principles to influence system credibility were limited to offering a level of surface credibility (n=15) (ie, “Do not show advertisements” and “At a first glance seem to offer truthful information”) and perceived expertise (ie, “expertise in the information provided”) (n=12). Some CA health apps also implemented verifiability (n=8) allowing users to find out more by linking to studies or reports that provide evidence, by providing real-world feel (highlighting the people and organizations behind the app, n=7), or by providing authority figures (medical doctors, n=7). Third-party endorsements were completely lacking.

As for the task support category, the most prevalent principle was to “Provide general information” (n=16). Other common task support principles were self-monitoring (being able to [re]view your own data) (n=12) and reduction (reducing complex behavior into simple tasks) (n=9). All of these apps offered reduction as the calculation of the Disease Activity Score (DAS) and visualized the evolution of the DAS score over time, hence allowing for self-monitoring. Goal setting in the form of setting specific goals and guiding the user towards that goal was less prevalent (n=3). Macrotailoring (tailoring information to specific patient groups) was found in only two apps, and finally both simulation and the use of contextual cues was found in only one app.

Dialogue support was present in the shape of reminders (n=10), mainly reminding patients to exercise or to fill out scores related to CA parameters, for example, number of sore joints, overall pain. One application also provided a reward in the shape of stars that could be collected when inputting information regularly. All other principles were lacking.

Finally, social support principles were less applied, in fact techniques to, for example, influence normative beliefs or to include provide social support were simply lacking in all the apps. Only one app implemented a means for social interaction by providing a link to a community forum where patients could exchange thoughts and feelings. Complete results can be found in Table 2 or Multimedia Appendix 1.

### User Ratings, Number of User Ratings, and Number of Installs

The average rating of all eligible apps (14/28, 50%) was 4.06 (SD 0.67) with a median 4.2 (out of 5). As mentioned, this average does not include apps that have fewer than five user ratings to minimize the influence of biased ratings. However, this distribution is negatively skewed (skewness 1.90).

On average (again excluding apps that did not meet the minimum of five user ratings), apps received 52 user ratings (SD 87), median 43, with the maximum number of user ratings recorded being 375. The distribution is heavily positively skewed (skewness 2.80). Only two apps have received +100 user ratings: RheumaTrack and RheumaHelper. RheumaTrack was developed with support from AbbVie, a company focused on pharmaceutical research and development and offers extensive logging of disease-related parameters. RheumaHelper was developed by an independent developer and offers a wide variety of calculations related to arthritis suitable for both patients and medical personnel.

**Figure 3:**
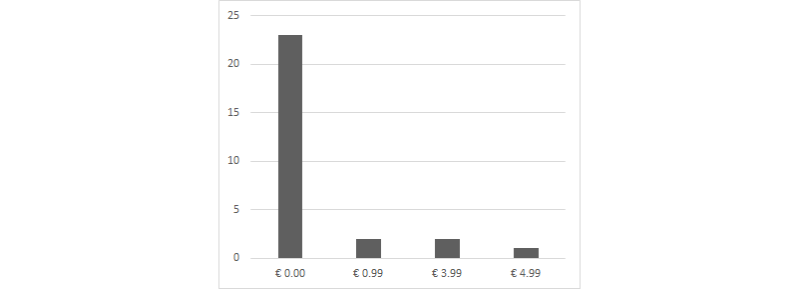
Number of apps per price category (N=28).

Finally, the average price was €0.57 (SD €1.45), with a median of €0.00. Of 28 apps, 23 were free. The highest price was €4.99. Figure 3 displays the frequency distribution of apps according to price category.

**Figure 4:**
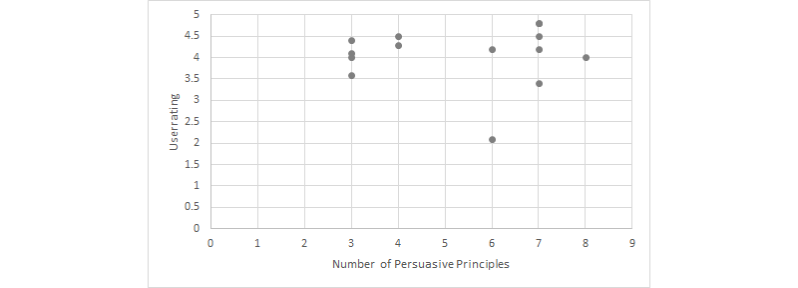
Number of persuasive principles in relation to user rating (n=14).

No significant correlation was found between the number of PSD principles and the number of user ratings and no significant correlation between the number of persuasive principles and average user rating. Figure 4 illustrates this relation between the number of persuasive principles and user rating.

Also, no significant correlation could be found between number of PSD principles and number of installs (including only the apps of Google Play Store).

## Discussion

### Principal Findings

The aim of this review was to study which persuasive principles were used in health apps and specifically in apps for CA patients. In the 28 apps we coded, we found a median of 6 persuasive principles, with most prominent categories being system credibility (median of 2 principles) and task support (median of 2 principles). Surprisingly, social support principles and dialogue support principles were lacking.

High system credibility implies that the apps are perceived as credible and trustworthy. Most of the apps that scored high on system credibility were either developed or funded by health services or pharmaceutical companies. Although the system credibility category is the most prevalent in our study, we argue that scoring only 2.6 (median 2) out of 7 possible system credibility principles per app is still surprisingly low. The first system credibility principle is surface credibility, characterized by the absence of obvious banners/ads. This was found in 26 apps (N=28). In addition, 25 apps equally offered expertise with respect to information about CA they presented. We argue that these principles are “low hanging fruit.” All CA apps, as all health apps, should use truthful information that is scientifically proven. Unfortunately, only 8 apps scored on verifiability (ie, the principle that implies a way to verify the content of the app). Third-party endorsements were completely lacking. Furthermore, information on system credibility was often available only when obtaining and installing the app.

With the open spirit of app stores, anyone can develop an app and publish it without having to provide evidence on its disease management tactics. This results in a substantial number of apps that seem to provide only a copy of static webpage content app format. It is remarkable that several apps were designed several years ago as confirmed by the last update date on their page on the app stores and seemed abandoned without any support from the developers. When browsing in the app store, it is not immediately clear which apps contain scientifically proven content and which do not. In line with the claim of Vollmer et al with “Apps seeking theories” [50], we argue that more apps need to give patients the means to verify the extent to which apps incorporate evidence-based practices.

The second highest prevalent category is task support (an average of 2.3 techniques per app with a median of 2). Task support lowers the effort for users to execute the target behavior by simplifying the task. In this study, apps mainly supported the task by providing more information on disease management or by providing a means to calculate the DAS score. In fact, many apps (11/28, 39%) could actually be classified as a “tracker” apps [55], that is, able to track a patient’s disease throughout a timespan of multiple days. Hence, trackers focus on being able to input disease data (either through manual input or automatic collection of data) and visualizing that data. By creating a place where patients can collect and visualize all their data, obviously “tracker” apps lower the effort for patients to manage their disease. However, the apps reviewed in this study, despite the advanced sensing and hardware opportunities that mobile phones offer, limited tracking to manual input. CA patients still had to manually enter a score about their physical activity level, rather than this being sensed through activity sensors inside the phone. In the authors’ opinion, many of the features that fitness and sports apps offer are simply not implemented in the CA apps reviewed in this study. In our humble opinion, much improvement can be made.

Dialogue support principles were implemented in only 10 apps (an average of 0.5 techniques per app with a median of 0). This category contains techniques that focus on feedback from the app to the patient. The most used technique we found in this category was to offer reminders. This is no surprise as patients need to take medication at regular intervals and reminders help them achieve that goal.

We note that dialogue support is a key feature of the more commercially successful fitness and sports apps like Nike+, Runkeeper, or Fitbit. Users can achieve a high score in this category through their use of rewards (commonly implemented as badges, trophies, points [56]), praise (motivational messages), and reminders. These so-called gamification techniques are currently popular in many apps [57-59] but lacking in CA apps, although health apps for CA patients may equally benefit from these techniques to motivate patients. Hence, we argue that CA apps could again benefit from implementing rewards and praise.

The least used category was social support; in fact, principles of this category were completely lacking. Again, this is in contrast with the aforementioned fitness and sports app and contrary to studies stating the importance of social support as mentioned by Kelders et al and others [44,60,61]. These apps include an extensive use of social support techniques to allow users to share their results and current activity on social media. It has been argued by many theories and researchers that, in particular, principles from social psychology may be effective in the long run. For example, relatedness has been linked to intrinsic motivation [62], normative beliefs have shown crucial for planned behavior change [43,63-65], and so on. Although social networks specifically for patients with chronic diseases exist [66], these social support techniques have not been used in any of the apps we reviewed. Again, we argue that particularly in this realm, health apps for CA patients can benefit from employing social support techniques.

To summarize, on the basis of our review, we argue that current health apps for CA patients would benefit from (1) a more evidence-based approach, by using content that can be verified as scientifically proven and endorsed by third parties, which in turn will result in an increase in system credibility, (2) the addition of automated tracking of certain health-related parameters (eg, physical activity, step count) that could further reduce the effort needed to manage a disease and thus increase task support. The current technology incorporated in mobile phones enables us to easily implement this functionality, (3) the extension of dialogue support techniques (eg, rewards, praise), in other words, the gamification principles also found in fitness and wellness apps, and (4) the addition of social support techniques (eg, social media, user forums).

### Limitations and Further Work

Unfortunately, we were not able to link the number of persuasive principles to patient appreciation. We found no correlation with user ratings and no correlation with number of installs. Perhaps there is simply no correlation, but this might also be attributed to our coding scheme. As stated by Lehto et al [45], the current PSD model, while highly valuable, is still evolving. Not much research has been done to validate its workings for assessing health apps, let alone health apps for CA patients in particular. For example, the current taxonomy does not include weights or priorities for the different techniques with respect to the management of CA. Yet, it is highly likely that not all persuasive principles are weighted as equally important by the CA patients. In particular, persuasive principles that lend themselves to long-term engagement may be more appropriate (eg, principles such as general information and goal setting [67,68]) than other principles that are associated more with short-term rewards (eg, reminders) [44,69]. Moreover, comorbidity of depression and chronic pain may suggest the importance of coping principles such as suggestion, social facilitation, and cooperation. Further studies may dissect which persuasive principles are deemed as more important by CA patients.

Moreover, some categories overlap, or remain elusive. Similar to Lehto et al [49], we found it hard to code some of the persuasive principles. Some principles are not hard grained as a feature of the app, but rather rely on an interpretation by the patient. An example of such a subjective principle is “Liking” from the PSD model. This principle is described as “A system that is visually attractive for its users” [32]. While this is a valid principle to increase persuasiveness, it can be argued that researchers are unable to judge whether certain apps are perceived as visually attractive by certain patient groups on the basis of the app alone. Other principles overlap, that is, one principle directly implied the use of the other. For example, an app that lacks surface credibility will not be perceived as trustworthy either. Moreover, the current persuasive principles are not specifically geared towards health or mobile apps. Hence, a taxonomy of persuasive principles geared towards mHealth apps is needed as well as further work on persuasive principles tailored for CA patients.

### Conclusion

In this study, we investigated which persuasive principles are most prevalent in current health apps targeted at CA patients. We found a remarkable lack of persuasive techniques to engage patients in digital management of their disease. Although persuasive principles such as social support and dialogue support contribute to the success of most fitness apps, health apps for chronic arthritis patients rarely use them. Several persuasive principles remain unused, leaving opportunities open to support patients in self-managing the disease. In particular, health apps for CA patients would benefit from adding social support techniques (eg, social media, user forums) and extending dialogue support principles (eg, rewards, praise). Tracking of certain health-related parameters (eg, physical activity, step count) could further reduce the effort needed to manage a disease and thus increase support. This knowledge might inform and inspire developers and health professionals to create apps that move beyond the current state and result in more motivating mHealth apps. Finally, health apps could certainly benefit from a more evidence-based approach, in offering content that can be verified as scientifically proven and endorsed by third parties.

## Appendix

Multimedia Appendix 1 Coding table.

## Abbreviations

BCT: behavior change technique
CA: chronic arthritis
DAS: Disease Activity Score
OA: osteoarthritis
PSD: Persuasive Systems Design
RA: rheumatoid arthritis
SpA: spondyloarthritis
