# Supplementary material for: A Review of Persuasive Principles in Mobile Apps for Chronic Arthritis Patients: Opportunities for Improvement
Source: JMIR Mhealth Uhealth. 2016 Oct 13;4(4):e118. doi: 10.2196/mhealth.6286 (PMC5083846; doi:10.2196/mhealth.6286)
Supplement: Multimedia Appendix 1 [file mhealth_v4i4e118_app1.pdf]

Appendix 1: Complete coding table

	Reduction	Goal Setting	Tunneling	Macro Tailoring	Micro Tailoring	Self-Monitoring
Andar	0	1	0	0	0	1
Arthritis	0	0	0	0	0	0
Arthritis Diary	1	0	0	0	1	1
Arthritis Relief	0	0	0	0	0	0
Arthritis Symptoms + Treatment	0	0	0	0	0	1
ArthritisID	1	0	0	1	0	1
Back to Action	0	0	0	0	0	0
Bewegen met Bechterew	0	0	0	0	1	0
DAS Calculadora	1	0	0	0	0	0
DAS28 - Rheumatoid Arthritis	1	0	0	0	0	0
DAS28 Free	1	0	0	0	0	0
iAnkylosing Spondylitis	0	0	0	0	0	0
Juvenile Rheumatoid Arthritis	0	0	0	0	0	0
Living Well With Arthritis	0	0	0	0	0	0
MyRA	0	0	0	0	0	1
NHS 24 MSK help	0	0	0	0	1	0
Pauseboogie fra Gigtforeningen	0	0	0	1	0	0
RA Helper	0	1	0	0	0	1
RADAI	1	0	0	0	0	0
RAPA – RA betegalkalmazás	1	0	0	0	0	1
Rheuma AKTIV	0	0	0	0	0	1
RheumaHelper	1	0	0	0	0	0
RhEumAtic Disease activitY	1	0	0	0	0	1
Rheumatoid Arthritis (RA) Patient Companion	0	0	0	0	0	1
Rheumatoid Arthritis Disease	0	0	0	0	0	0
Rheumatoid Arthritis of Knee	0	0	0	0	0	0
RheumaTrack® RA	1	0	0	0	0	1
Track + React	0	1	0	0	0	1

Praise	General Information	Behavioral Contract	Contextual Cues	Rehearsal	Instruction	Simulation	Logging	Tracking
0	0	0	0	0	0	0	1	0
0	1	0	0	0	0	0	0	0
0	0	0	1	0	0	1	1	0
0	1	0	0	0	0	0	0	0
0	1	0	0	0	0	0	0	0
0	1	0	0	0	0	0	1	0
0	0	0	0	0	1	0	0	0
0	0	0	0	0	1	0	0	0
0	1	0	0	0	0	0	0	0
0	0	0	0	0	0	0	0	0
0	0	0	0	0	0	0	0	0
0	1	0	0	0	1	0	0	0
0	1	0	0	0	0	0	0	0
0	1	0	0	0	0	0	0	0
0	0	0	0	0	0	0	1	0
0	1	0	0	0	1	0	0	0
0	0	0	0	0	1	0	0	0
0	0	0	0	0	0	0	1	0
0	1	0	0	0	0	0	0	0
0	1	0	0	0	1	0	1	0
0	1	0	0	0	1	0	0	0
0	0	0	0	0	0	0	0	0
0	0	0	0	0	0	0	0	0
0	1	0	0	0	0	0	1	0
0	1	0	0	0	0	0	0	0
0	1	0	0	0	0	0	0	0
0	0	0	0	0	0	0	1	0
0	1	0	0	0	0	0	1	0

Verifiability	Expertise	Surface credibility	Prompt Selftalk	Social role	Personalization	Similarity	Suggestion	Reminders	Rewards
0	1	1	0	0	0	0	0	1	0
0	0	0	0	0	0	0	0	0	0
0	1	1	0	0	0	0	0	1	0
0	0	1	0	0	0	0	0	0	0
0	0	1	0	0	0	0	0	0	0
0	1	1	0	0	0	0	0	1	0
1	1	1	0	0	0	0	0	0	0
0	1	1	0	0	0	0	0	0	0
1	1	1	0	0	0	0	0	0	0
0	0	1	0	0	0	0	0	0	0
0	0	1	0	0	0	0	0	0	0
1	1	1	0	0	0	0	0	0	0
0	1	1	0	0	0	0	0	0	0
0	1	1	0	0	0	0	0	0	0
0	1	1	0	0	0	0	0	1	0
1	1	1	0	0	0	0	0	1	0
1	1	1	0	0	0	0	0	1	0
0	1	1	0	0	0	0	0	1	0
1	1	1	0	0	0	0	0	0	0
0	1	1	0	0	0	0	0	0	0
1	1	1	0	0	0	0	0	1	1
1	1	1	0	0	0	0	0	0	0
0	1	1	0	0	0	0	0	0	0
1	1	1	0	0	0	0	0	1	0
0	1	1	0	0	0	0	0	0	0
0	1	0	0	0	0	0	0	0	0
0	1	1	0	0	0	0	0	1	0
0	1	1	0	0	0	0	0	0	0

Normative influence	Social comparison	Social Identification	Social learning	Third-party endorsements	Authority	Real-world feel
0	0	0	0	0	0	0
0	0	0	0	0	0	0
0	0	0	0	0	0	0
0	0	0	0	0	0	0
0	0	0	0	0	0	0
0	0	0	0	0	1	1
0	0	0	0	0	1	1
0	0	0	0	0	1	1
0	0	0	0	0	0	0
0	0	0	0	0	0	0
0	0	0	0	0	0	0
0	0	0	0	0	0	1
0	0	0	0	0	0	0
0	0	0	0	0	0	0
0	0	0	0	0	1	0
0	0	0	0	0	1	1
0	0	0	0	0	0	1
0	0	0	0	0	0	0
0	0	0	0	0	0	0
0	0	0	0	0	0	0
0	0	0	0	0	0	0
0	0	0	0	0	0	0
0	0	0	0	0	0	0
0	0	0	0	0	1	1
0	0	0	0	0	0	0
0	0	0	0	0	0	0
0	0	0	0	0	0	0
0	0	0	0	0	1	0

**Social facilitation**	0	0	0	0	0	0	0	0	0	0	0	0	0	0	0	0	0	0	0	0	0	0	0	0	0	0	0	0
**Cooperation**	0	0	0	0	0	0	0	0	0	0	0	0	0	0	0	0	0	0	0	0	0	0	0	0	0	0	0	0
**Competition**	0	0	0	0	0	0	0	0	0	0	0	0	0	0	0	0	0	0	0	0	0	0	0	0	0	0	0	0
**Recognition**	0	0	0	0	0	0	0	0	0	0	0	0	0	0	0	0	0	0	0	0	0	0	0	0	0	0	0	0
**Social Interaction**	0	0	0	0	0	1	0	0	0	0	0	0	0	0	0	0	0	0	0	0	0	0	0	0	0	0	0	0
